# Plasma microRNA Profiling Reveals Novel Biomarkers of Epicardial Adipose Tissue: A Multidetector Computed Tomography Study

**DOI:** 10.3390/jcm8060780

**Published:** 2019-06-01

**Authors:** David de Gonzalo-Calvo, David Vilades, Pablo Martínez-Camblor, Àngela Vea, Andreu Ferrero-Gregori, Laura Nasarre, Olga Bornachea, Jesus Sanchez Vega, Rubén Leta, Núria Puig, Sonia Benítez, Jose Luis Sanchez-Quesada, Francesc Carreras, Vicenta Llorente-Cortés

**Affiliations:** 1 Institute of Biomedical Research of Barcelona (IIBB) - Spanish National Research Council (CSIC), 08036 Barcelona, Spain; olgabornachea79@gmail.com; 2 Biomedical Research Institute Sant Pau (IIB Sant Pau), 08041 Barcelona, Spain; angelavea@gmail.com (À.V.); lnasarre@santpau.cat (L.N.); 3 CIBERCV, Institute of Health Carlos III, 28029 Madrid, Spain; AFerrero@santpau.cat (A.F.-G.); FCarreras@santpau.cat (F.C.); 4 Cardiac Imaging Unit, Cardiology Department, Hospital de la Santa Creu i Sant Pau, 08041 Barcelona, Spain; DVilades@santpau.cat (D.V.); JSanchezV@santpau.cat (J.S.V.); RLeta@santpau.cat (R.L.); 5 Geisel School of Medicine, Dartmouth College, Hanover, NH 03755, USA; pablo.martinez.camblor@dartmouth.edu; 6 Cardiology Service, Hospital de la Santa Creu i Sant Pau, Universitat Autònoma de Barcelona (UAB), 08041 Barcelona, Spain; 7 Cardiovascular Biochemistry, Biomedical Research Institute Sant Pau (IIB Sant Pau), 08041 Barcelona, Spain; NPuigG@santpau.cat (N.P.); SBenitez@santpau.cat (S.B.); JSanchezQ@santpau.cat (J.L.S.-Q.); 8 Molecular Biology and Biochemistry Department, Universitat Autònoma de Barcelona (UAB), 08193 Cerdanyola del Valles, Spain; 9 CIBERDEM, Institute of Health Carlos III, 28029 Madrid, Spain

**Keywords:** biomarker, cardiometabolic disease, epicardial adipose tissue, epicardial fat, epicardial fat volume, microRNA

## Abstract

Epicardial adipose tissue (EAT) constitutes a novel parameter for cardiometabolic risk assessment and a target for therapy. Here, we evaluated for the first time the plasma microRNA (miRNA) profile as a source of biomarkers for epicardial fat volume (EFV). miRNAs were profiled in plasma samples from 180 patients whose EFV was quantified using multidetector computed tomography. In the screening study, 54 deregulated miRNAs were identified in patients with high EFV levels (highest tertile) compared with matched patients with low EFV levels (lowest tertile). After filtering, 12 miRNAs were selected for subsequent validation. In the validation study, miR-15b-3p, miR-22-3p, miR-148a-3p miR-148b-3p and miR-590-5p were directly associated with EFV, even after adjustment for confounding factors (*p* value < 0.05 for all models). The addition of miRNA combinations to a model based on clinical variables improved the discrimination (area under the receiver-operating-characteristic curve (AUC) from 0.721 to 0.787). miRNAs correctly reclassified a significant proportion of patients with an integrated discrimination improvement (IDI) index of 0.101 and a net reclassification improvement (NRI) index of 0.650. Decision tree models used miRNA combinations to improve their classification accuracy. These results were reproduced using two proposed clinical cutoffs for epicardial fat burden. Internal validation corroborated the robustness of the models. In conclusion, plasma miRNAs constitute novel biomarkers of epicardial fat burden.

## 1. Introduction

MicroRNAs (miRNAs) are evolutionarily conserved small noncoding RNAs (ncRNAs) that posttranscriptionally regulate gene expression playing a critical role in cellular pathways involved in development, homeostasis and the response to stress [1]. In addition to their intracellular localization, miRNAs have also been detected in the extracellular space and circulation [2]. Extracellular miRNAs can be easily assayed by analyzing bodily fluids, they are stable against degradation, have a long half-life in biological samples, and can be detected with techniques readily available in clinical laboratories [3]. Thus, miRNA-based tests provide an interesting opportunity to develop novel tools to assist in clinical decision-making [3]. A number of publications have proposed the use of circulating miRNAs as biomarkers for a wide range of medical conditions [4,5]. Indeed, monitoring alterations in the patterns of circulating miRNAs could be useful in the diagnosis and prognostic stratification within diverse cardiovascular and metabolic disorders [6,7].

Epicardial adipose tissue (EAT), the visceral fat depot located between the myocardium and visceral pericardium, is a metabolically active tissue that regulates cardiovascular homeostasis under physiological conditions [8]. However, under pathological conditions, excessive accumulation of EAT surrounding the myocardium and the coronary arteries actively contributes to the development and progression of cardiovascular disease [9]. Although the mechanisms are not fully understood, EAT has been directly implicated in different pathological processes via secretion of pro-inflammatory, pro-fibrotic and metabolic mediators [10,11]. The relationship between epicardial fat burden and cardiovascular disease has recently gained attention among the medical community. Multiple clinical studies have demonstrated that epicardial fat is a risk factor for coronary artery disease (CAD) [12], heart failure (HF) [13] and atrial fibrillation (AF) [14]. A prognostic value for major adverse cardiovascular events and mortality has also been reported [15]. Furthermore, epicardial fat accumulation has been correlated with metabolic diseases including diabetes and metabolic syndrome [16]. Consequently, EAT constitutes a novel parameter for cardiometabolic risk assessment and a target for therapy [17,18].

Epicardial adipose tissue can be clinically measured using different imaging techniques such as transthoracic echocardiography, cardiovascular magnetic resonance (CMR) and computed tomography (CT). However, the need for specialized centers and trained personnel, in addition to the operating expenses, limit the applicability of this methodology. Surprisingly, there are limited studies investigating non-invasive and easily accessible biomarkers to quantify EAT [19,20]. Despite the potential of miRNAs as clinical indicators, they have not yet been used to assess the epicardial fat burden. Here, we address this important gap by evaluating the plasma miRNA profile to identify biomarkers for epicardial fat volume (EFV).

## 2. Experimental Section

### 2.1. Study Population

This is a prospective study including clinically stable patients referred for coronary computed tomography angiography (CCTA) in the Cardiac Imaging Unit of the Hospital de la Santa Creu i Sant Pau (Barcelona, Spain). According to miRNA profiling data, accepting a significance level of 0.05 and a power of 80% in a two-sided test, and assuming a common standard deviation (SD) of 0.9, 51 subjects were necessary in both study groups to recognize as statistically significant a difference greater than or equal to 0.5 arbitrary units (au). EAT assessment was performed after blood collection. Therefore, the sample size used was higher to ensure the necessary number of patients. Finally, 180 consecutive patients were enrolled in the study. Exclusion criteria included suspected acute coronary syndrome, contraindications to CCTA imaging, any survival-limiting disease or any severe infectious disease. Detailed demographic, anthropometric, clinical and pharmacological information was obtained from electronic medical records. All subjects gave written informed consent before participating in the study. The study protocol was approved by the local ethical committee of the Hospital de la Santa Creu i Sant Pau. The study was performed in accordance with the Helsinki Declaration.

### 2.2. Coronary Computed Tomography Angiography (CCTA)

A CCTA exam using a 256-slice CT scanner (Brilliance iCT 256; Philips Healthcare, Amsterdam, the Netherlands) was performed on all participants. A contrast-enhanced scan was performed to assess CAD and EFV. The scan was prospectively triggered at 75% of the RR interval using 100 kV (120 kV in patients with a body mass index >30 kg m^−2^) if the heart rate was below 65 beats per min (bpm), and retrospectively gated (helical acquisition) if the heart rate was higher than 65 bpm. Iodinated contrast (Xenetix 350; Guerbet, Aulnay-sous-Bois, France) was administered at a dose of 0.7–1 mL kg^−1^ (range 50–120) and followed by a 40 mL saline flush, and both injected at a rate of 5–6 mL/s. CCTA studies were subsequently analyzed off-line. Coronary artery plaques were defined as any tissue structure >1 mm^2^ that existed either within the coronary artery lumen or adjacent to the coronary artery lumen that could be distinguished from the surrounding pericardial tissue, epicardial fat, or the lumen itself. Coronary artery disease was quantified for stenosis by quantitative coronary angiography (CT-QCA) in any luminal diameter narrowing ≥50% of the reference luminal diameter. The methodology to calculate EFV was performed with a dedicated software as follows (OsiriX MD, v 6.5, FDA cleared, Pixmeo): first, the upper and lower slice limits of pericardium were manually defined in an axial view. Then, the EFV was marked in each slice by drawing regions of interest with voxel densities between −150 to −30 Hounsfield units (corresponding to adipose tissue). A contiguous 3-dimensional volume render was then performed and quantified in cubic centimeters (cm^3^), and indexed to body surface area (cm^3^ m^−2^) to produce an EFV-index (EFVi). Body surface area data was available for 160 patients. Patients were stratified according EFV tertiles: first and second tertiles (11.93–118.00 cm^3^) and third tertile (118.74–257.35 cm^3^). Additionally, we used two binary cutoffs previously proposed as clinically relevant: EFV > 125 cm^3^ [15] and EFVi > 68.1 cm^3^ m^−2^ [21].

### 2.3. Blood Collection

Blood collection and processing were performed using standardized protocols [22]. Blood samples were obtained in K_2_-ethylenediaminetetra-acetic acid (EDTA) blood collection tubes (BD) by venipuncture after a night of fasting and before beginning any interventional procedure or administration of contrast agents. The blood was processed within 2 h after isolation. To obtain plasma, blood samples were fractionated by centrifugation at 1300 × g for 15 min at room temperature. After centrifugation, plasma supernatant was aliquoted into 1.5 mL DNA LoBind tubes and stored at −80 °C until analysis.

### 2.4. Epicardial Adipose Tissue (EAT)

Epicardial adipose tissue explants were obtained from patients undergoing cardiac surgery (*N* = 8). Patients were diagnosed with either CAD (*N* = 4) or valve disease (*N* = 4). 100 mg pieces of EAT were incubated for 24 h in 1 mL serum-free DMEM supplemented with antibiotics in 5% CO_2_. miRNAs were isolated from tissue and from the conditioned media.

### 2.5. High-Sensitive C-Reactive Protein (CRP) Concentration

High-sensitive C-reactive protein (hs-CRP) concentrations were determined using an immunoturbidimetry method on the Roche Cobas c501 analyzer (Roche Diagnostics, Mannheim, Germany). The hs-CRP assay has an analytic range from 0.3 to 350 mg L^−1^. The assay had interrun coefficients of variation that ranged from 1.2 to 3.6%.

### 2.6. MicroRNA Isolation

Profiling of miRNA was conducted in the same laboratory and under the same conditions. Experienced staff blinded to clinical data performed all laboratory measurements. Total RNA was isolated from 150 μL of frozen plasma samples or conditioned medium samples using miRNeasy Serum/Plasma Kit (Qiagen, Hilden, Germany), according to the manufacturer’s instructions. For EAT, total RNA was isolated from 100 mg of tissue using the miRNeasy Mini Kit (Qiagen). For normalization of extracellular miRNAs, synthetic *Caenorhabditis elegans* miR-39-3p (cel-miR-39-3p), lacking sequence homology to human miRNAs, was added as an external reference miRNA (1.6 × 10^8^ copies μL^−1^). The mixture was also supplemented with 1 µg of MS2 carrier RNA (Roche, Merck, Darmstadt, Germany) to improve extracellular miRNA yield. Purification of RNA was performed with RNeasy MinElute or RNeasy Mini Spin columns according to the manufacturer’s instructions. RNA was eluted in nuclease-free H_2_O and stored in a −80 °C freezer.

### 2.7. Quantification of MicroRNA

Quantitative polymerase chain reaction (qPCR) was performed according to the protocol for the miRCURY LNA Universal RT microRNA PCR System (Exiqon, Qiagen, Hilden, Germany), which offers an optimal performance [23]. According to the manufacturer’s instructions, different protocols for cDNA synthesis were used for extracellular or tissue miRNAs. The RNA in the plasma and conditioned media cannot be accurately quantified. Therefore, we used the same starting sample volume rather than RNA quantity (2 µL of RNA). For tissue, total RNA concentration was determined with a NanoDrop ND-1000 spectrophotometer (NanoDrop Technologies). Then, RNA samples were adjusted to a concentration of 5 ng μL^−1^ using nuclease-free H_2_O. RNA was reverse transcribed in 10 µL reactions using the Universal cDNA Synthesis Kit II (Exiqon). The RT reaction was performed with the following conditions: incubation for 60 min at 42 °C followed by heat-inactivation for 5 min at 95 °C; the reaction was then immediately cooled to 4 °C. cDNA was stored at −20 °C.

For the screen, we used the 384 well Serum/Plasma Focus microRNA PCR Panel V4 (Exiqon). The panel included primer sets for 179 miRNAs commonly found in serum and plasma samples. Each selected miRNA was validated in plasma, conditioned media and tissue using 384-well Pick-&-Mix microRNA PCR Plates (V4) (Exiqon). qPCR was performed in 10 µL reactions using the 7900HT Fast Real-Time PCR System (Applied Biosystems, Thermo Fisher Scientific, Waltham, Massachusetts, USA) with the following cycling conditions: 10 min at 95 °C, 40 cycles of 10 s at 95 °C and 1 min at 60 °C, followed by a melting curve analysis. The synthetic UniSp3 assay was analyzed as interplate calibrator. The SDS v2.3 software was used to determine the quantification cycle number (Cq) and perform the melting curve analysis. The Cq was defined as the fractional cycle number at which the fluorescence exceeded a given threshold. The specificity of the qPCR was corroborated by melting curve analysis. miRNAs were considered to be expressed at Cq values < 35. Relative quantification was performed using the 2^−dCq^ method, where dCq = Cq_miRNA_ − Cq_cel-miR-39-3p_ for extracellular miRNAs and Cq_miRNA_ − CqSNORD48 for tissue miRNAs. Expression levels were log_2_-transformed for statistical analyses. 

### 2.8. Statistical Analysis

Statistical analysis was performed using the statistical software package R version 3.5.2. Descriptive statistics were used to summarize the characteristics of the study population. The Kolmogorov–Smirnov test was used to test normality. Data were described as the mean ± SD and median (P25–P75) for continuous variables. Frequency (percentage) was used for categorical variables. Continuous variables were compared between groups using the Student’s *t*-test and Mann–Whitney U test for normally distributed and nonnormally distributed variables, respectively. Categorical variables were compared between groups using Fisher’s exact test. Spearman’s rho coefficient was used to assess the correlation between continuous variables. In the screen, heat map visualization was used to determine whether plasma miRNAs can differentiate between patients according to EFV tertile [24]. In the validation study, logistic regression analyses were used to investigate whether plasma miRNAs were independently associated with EFV.

Backward stepwise regression models were used to explore the performance of plasma miRNAs, in combination with clinical covariates, as biomarkers of EFV. Clinical covariates were chosen based on statistical differences observed between study groups (*p* value < 0.1): age, sex, body mass index (BMI) and diabetes mellitus. The results were presented as an odds ratio (OR) and 95% confidence intervals (CI). Receiver-operating-characteristic (ROC) curves were constructed to assess the global discriminative ability. The results were presented as the area under the ROC curve (AUC) and 95% CI. The added discrimination capacity of plasma miRNA over the multiparameter clinical model was tested by the DeLong test [25]. The Integrated Discrimination Improvement (IDI) index and Net Reclassification Improvement (NRI) index were computed to assess the reclassification capacity of plasma miRNAs [26]. The internal validity of the final models was tested for 500 bootstrap resamples, using the ‘rms’ package by Frank Harrell [27] in the R Project for Statistical Computing. The calibration of the models was assessed by the corresponding plots using the same package.

Decision tree models were developed using a chi-squared automatic interaction detector (CHAID) algorithm [28]. The CHAID algorithm utilizes statistical significance from Chi-square tests to establish a hierarchy of predictors, here the parameters that composed our clinical model and plasma miRNAs. CHAID analysis identifies potential interactions among the predictors and selects the optimal combination of variables and cutoff values for classification. The two-tailed significance level was set at *p* value < 0.05.

## 3. Results

### 3.1. Study Population

Table 1 shows the characteristics of the study population. The mean age was 65.0 ± 12.8 years, and 104 patients (58%) were male. The prevalence for hypertension, dyslipidemia, diabetes mellitus and active or former smoker was 62%, 57%, 21% and 33%, respectively. Patients underwent multiple pharmacological therapies including antiplatelet drugs (41%), statins (48%), beta-blockers (32%), angiotensin-converting-enzyme (ACE) inhibitors (54%) and diuretics (27%).

Standardized quantitative categories for EFV are currently lacking. Therefore, the study population was stratified according to EFV tertiles: patients in the first and second tertiles (low-medium epicardial fat burden) and patients in the third tertile (high epicardial fat burden). Compared to patients in the first and second tertiles of EFV, patients in the third tertile were typically older, more frequently male with a higher BMI and prevalence of diabetes mellitus (Table 1). The use of statin, an antiplatelet drug, and beta-blockers was also higher in patients in the third tertile.

### 3.2. Profiling of Plasma MicroRNAs

**Figure 1:**
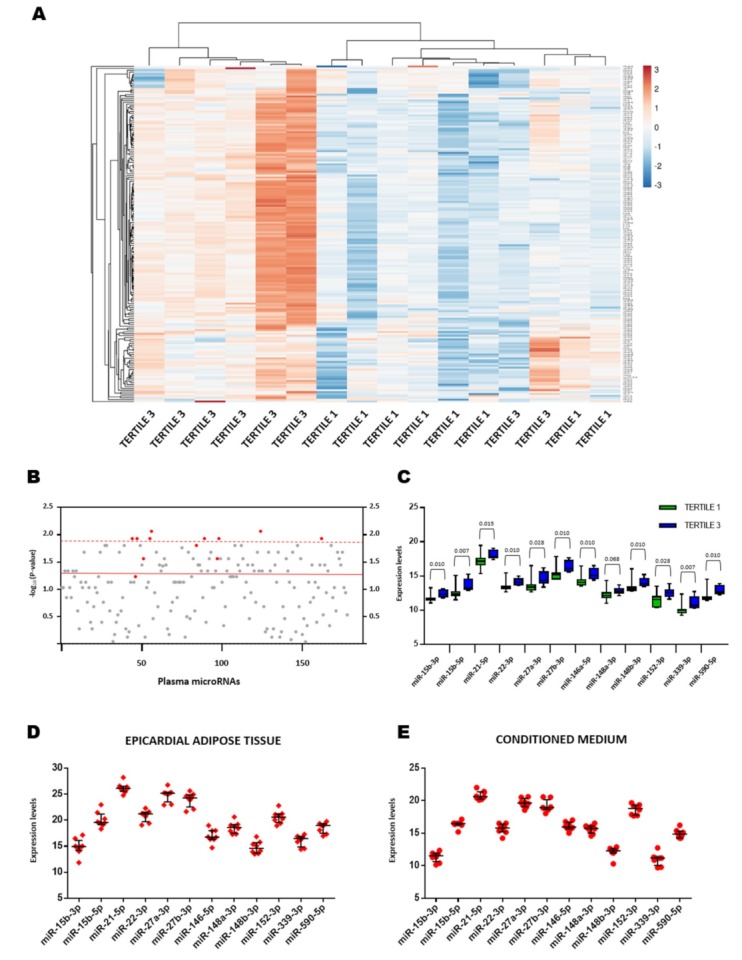
Plasma microRNA (miRNA) profiling. (**A**) Unsupervised hierarchical clustering. The heat map diagram shows the result of a two-way hierarchical clustering of patients and miRNAs. Each column represents a patient (Tertile 1 of epicardial fat volume vs. Tertile 3 of epicardial fat volume). Each row represents a miRNA. The patient clustering tree is shown on top. The miRNA clustering tree is shown on the left. The color scale illustrates the relative expression level of miRNAs. The expression intensity of each miRNA in each sample varies from red to blue, which indicates relatively high or low expression, respectively. (**B**) *p* value for the comparison between study groups. Each point represents a miRNA. Red dots represent the selected candidates. (**C**) Plasma expression levels of miRNAs in study groups. (**D**) Expression levels of miRNAs in epicardial adipose tissue explants. Each point represents a sample. (**E**) Expression levels of miRNAs in conditioned media exposed to epicardial adipose tissue explants. Each point represents a sample. Relative quantification was performed using cel-miR-39-3p as the external standard for extracellular miRNAs and SNORD48 as the internal standard for tissue miRNAs. MicroRNA levels were log2-transformed. MicroRNA expression levels are expressed as arbitrary units. Differences between groups were analyzed using the Mann–Whitney U test. *p* values describe the significance level of differences for each comparison.

To determine whether the plasma miRNAs were differentially expressed between study groups, we first profiled the expression of 179 miRNAs in patients with low (first EFV tertile, *N* = 8) and high epicardial fat burden (third EFV tertile, *N* = 8) (Table S1). Due to the sample size and in order to gain statistical power, patients in the second EFV tertile were not included in this phase. To minimize potential confounding variables, we also restricted our analysis to patients in the first and third tertiles who matched according to age, sex, BMI, cardiovascular risk factors and hs-CRP levels. The expression level of miR-208a-3p was below the limit of detection in 94% of the samples and was excluded from additional analyses. Unsupervised hierarchical clustering based on miRNA expression profile clearly separated patients in the third tertile from patients in the first tertile (Figure 1A). Analysis of the data identified 54 significantly differentially expressed miRNAs (Figure 1B, Table S2). To identify potential biomarkers, we selected 8 miRNAs (miR-15b-3p, miR-15b-5p, miR-22-3p, miR-27b-3p, miR-146a-5p, miR-148b-3p, miR-339-3p and miR-590-5p) for further validation based on their statistical significance (*p* ≤ 0.01) and abundance in the circulation (median Cq < 30, maximum Cq = 32 and detected in all samples) (Figure 1C, Table S2). Several differentially expressed miRNAs belonged to the same family as our candidates: miR-21-5p, miR-27a-3p, miR-148a-3p (*p* value = 0.065) and miR-152-3p (Figure 1C, Table S2). Members within the same miRNA family share seed sequences and could be functionally related (Table S3). Thus, these miRNAs were also selected for further validation in order to test whether the combination of all family members could have higher potential as biomarker than individual members. These miRNAs were all abundantly expressed in the circulation, meeting the established criteria. Except for miR-15b-3p and miR-15b-5p, all of the candidates were derived from different miRNA genomic clusters (>10 kb) (Table S3).

To further characterize the potential of these miRNAs as biomarkers of EFV, we evaluated their expression levels in human EAT explants and in conditioned media that was exposed to human EAT explants. The miRNA profiles were similar in both sample sets, and all of the miRNAs were detected in all tissue and conditioned media samples (Cq < 35) (Figure 1D,E).

### 3.3. Plasma MicroRNAs and Epicardial Fat Volume

**Figure 2:**
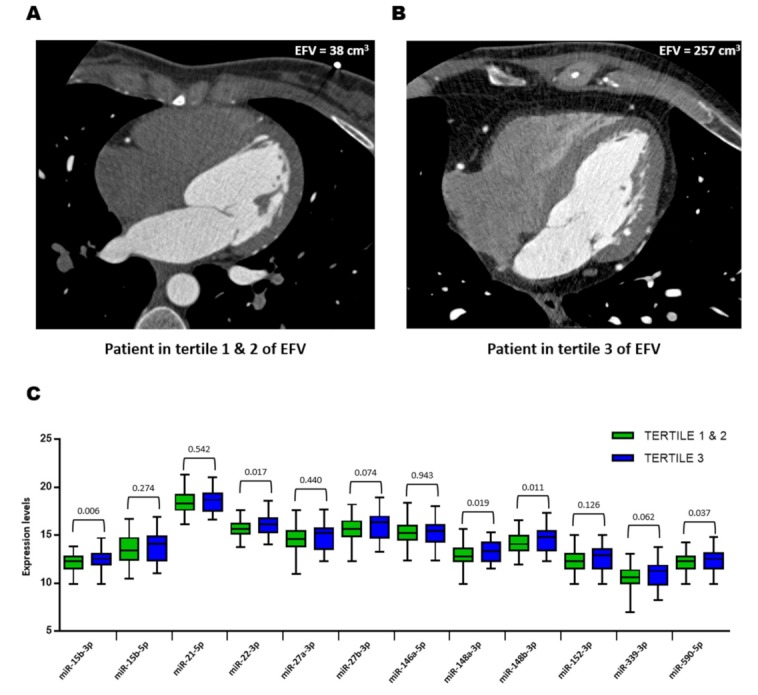
Plasma microRNA (miRNA) validation. (**A–B**) Examples of multidetector computed tomography scans and the corresponding epicardial fat volume of patients in the first-second and third tertiles of epicardial fat volume. (**C**) Plasma expression levels of miRNAs in study groups. MicroRNA levels were log_2_-transformed. MicroRNA expression levels are expressed as arbitrary units. Differences between groups were analyzed using Student’s *t*-test for independent samples. *p* values describe the significance level of differences for each comparison. EFV: epicardial fat volume.

Selected miRNAs from the screen were validated in the whole study population (*N* = 180). To do that, patients were stratified into two categories: low-medium epicardial fat burden (patients in the first and second EFV tertiles, *N* = 120) and high epicardial fat burden (patients in the third EFV tertile, *N* = 60). Representative patients in the first-second and third EFV tertiles are shown in Figure 2A,B. As shown in Figure 2C, the plasma expression levels of miR-15b-3p, miR-22-3p, miR-148a-3p, miR-148b-3p and miR-590-5p were significantly higher in patients in the third tertile compared with those in the first and second tertiles. Plasma miR-15b-3p, miR-22-3p, miR-148a-3p and miR-148b-3p were directly correlated with EFV (Table S4).

Logistic regression models were used to evaluate the associations between EFV (first and second tertiles vs. third tertile) and miRNAs (Table 2). Using unadjusted logistic regression models (model 1), the plasma levels of miR-15b-3p, miR-22-3p, miR-148a-3p, miR-148b-3p and miR-590-5p were directly associated with EFV. After correcting for confounding factors including age, sex, BMI, diabetes mellitus, medication use (antiplatelet drugs, statin use and beta-blockers use) and CAD (models 2, 3 and 4), the association between the EFV and these miRNAs remained statistically significant.

### 3.4. Performance of Plasma MicroRNAs as Biomarkers of Epicardial Fat Volume

**Figure 3:**
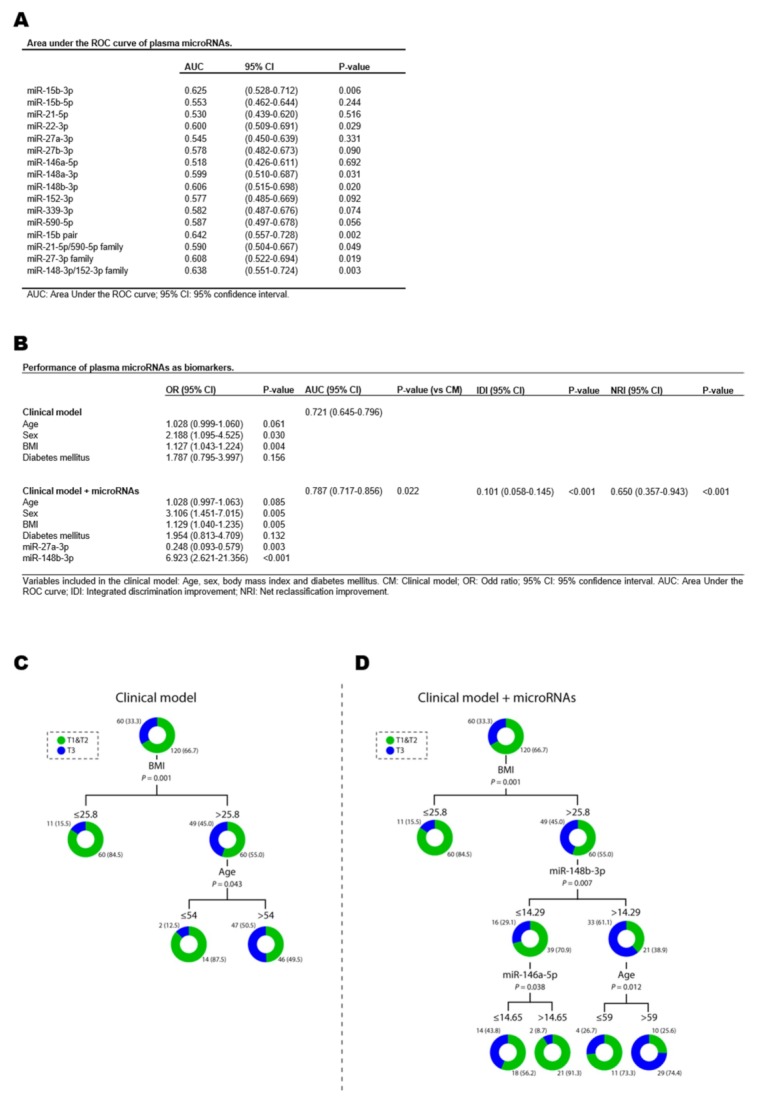
Plasma microRNAs (miRNAs) as biomarkers of epicardial fat volume, according to EFV tertiles. (**A**) Area under the ROC curve (AUC) for each individual miRNAs and for combinations of miRNAs in pairs or families. (**B**) Performance of plasma miRNAs as biomarkers. (**C**,**D**) Decision trees calculated by chi-squared automatic interaction detector (CHAID) algorithm. The following variables were included in the clinical model: age, sex, body mass index and diabetes mellitus. MicroRNA levels were log_2_-transformed. For logistic regression models, data are presented as an odds ratio (OR) and 95% confidence intervals (CI). For discrimination analysis, data are presented as the AUC and 95% CI. For reclassification analysis, data are presented as the Integrated Discrimination Improvement (IDI) index and Net Reclassification Improvement (NRI) index and their respective and 95% CI. For decision trees, data are shown as frequency (percentage) of patients in each study group.

The AUC was used to assess the discriminative capacity of plasma miRNAs as biomarkers for EFV. As shown in Figure 3A, all of the individual miRNAs showed poor discrimination ability (AUC = 0.518–0.625). The discrimination capacity was also modest for the combination of miRNAs in pairs or families (AUC = 0.590–0.642).

To further explore the role of plasma miRNAs as potential biomarkers for EFV, we evaluated the effect of adding our miRNAs on the discrimination capacity of a model originally based on clinical variables: age, sex, BMI and diabetes mellitus (clinical model). When the miRNAs were added as independent variables (clinical model + plasma miRNAs), miR-27a-3p and miR-148b-3p were significant predictors of EFV (Figure 3B). Comparison of ROC curves showed that the AUC for the clinical model + plasma miRNAs was significantly higher (9.2%) than that of the clinical model alone: AUC = 0.721 vs. 0.787. Adding both miRNAs to the clinical model also led to a significant reclassification of the patients: IDI = 0.101 and NRI = 0.650. Bootstrap internal validation supported the robustness of the model including plasma miRNAs (Figure S1A).

Decision tree models were constructed using the CHAID algorithm. First, we included the variables from the clinical model. As shown in Figure 3C, the decision tree identified the cutoff values for BMI and age. The first variable selected was BMI. For patients with a BMI >25.8 kg m^−2^, age was the next most relevant predictor with a cutoff value of 54 years. Second, in addition to the variables from the clinical model, we included the miRNA candidates (Figure 3D). Again, BMI was the first variable selected. In this case, miR-148b-3p was the next most significant predictor for patients with BMI >25.8 kg m^−2^. A cutoff expression value of 14.29 arbitrary units (au) allowed the enrichment in two subgroups of patients in the first and second tertiles (70.9%) and the third tertile (61.1%). In patients with a BMI >25.8 kg m^−2^ and miR-148b-3p expression levels ≤14.29 au, a miR-146a-5p expression level >14.65 au increased the percentage of patients in the first and second tertiles to 91.3%. In patients with BMI >25.8 kg m^−2^ and miR-148b-3p expression levels >14.29 au, age was the most significant predictor, increasing the percentage of patients older than 59 years that belonged to the third tertile to 74.4%.

### 3.5. Validation Using Alternative Cutoffs of Epicardial Fat Volume

**Figure 4:**
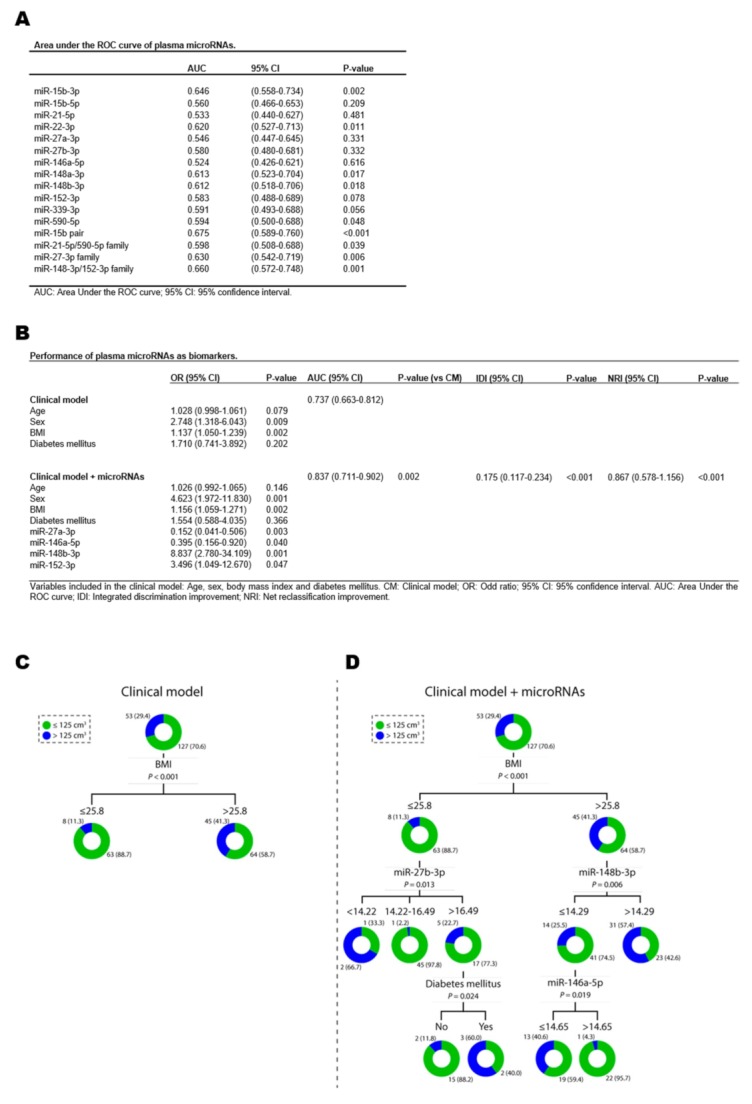
Plasma microRNAs (miRNAs) as biomarkers of epicardial fat volume, according to the cutoff values proposed by Spearman et al. [15]. (**A**) Area under the ROC curve (AUC) for each individual miRNAs and for combinations of miRNAs in pairs or families. (**B**) Performance of plasma miRNAs as biomarkers. (**C**,**D**) Decision trees calculated by Chi-squared Automatic Interaction Detector (CHAID) algorithm. The following variables were included in the clinical model: age, sex, body mass index and diabetes mellitus. MicroRNA levels were log_2_-transformed. For logistic regression models, data are presented as an odds ratio (OR) and 95% confidence intervals (CI). For discrimination analysis, data are presented as the AUC and 95% CI. For reclassification analysis, data are presented as the Integrated Discrimination Improvement (IDI) index and Net Reclassification Improvement (NRI) index and their respective 95% CI. For decision trees, data are shown as frequency (percentage) of patients in each study group.

To validate these findings, we evaluated the ability of our miRNAs to serve as biomarkers using previously published binary cutoffs of epicardial fat burden: Spearman et al. (EFV ≤125 cm^3^ vs. EFV >125 cm^3^) [15] (Figure 4) and Shmilovich et al. (EFVi ≤68.1 cm^3^ m^−2^ vs. (EFVi >68.1 cm^3^ m^−2^) [21] (Figure S2). The discrimination capacity was modest for all individual miRNAs and when combined in pairs or families for both clinical ranges (Figure 4A, Figure S2A). In support of the above findings, adding the candidates to the clinical models demonstrated that plasma miRNAs, in particular miR-27a-3p, miR-146a-5p, miR-148b-3p and miR-152-3p, were significant predictors of EFV and EFVi (Figure 4B, Figure S2B). The addition of miRNAs significantly augmented the discriminative power of the clinical models and reclassify a significant proportion of the patients (Figure 4B, Figure S2B). The robustness of the models including miRNAs was confirmed by bootstrap (Figure S1B,C).

The decision tree model identified several plasma miRNAs, miR-27b-3p, miR-146a-5p and miR-148b-3p for classification using the clinical range proposed by Spearman et al. (Figure 4C,D). miR-27a-3p and miR-339-3p were also selected using the clinical range proposed by Shmilovich et al. (Figure S2C,D).

## 4. Discussion

Our study provides the first insight into the value of the circulating miRNA signature as a source of clinical indicators for epicardial fat assessment.

First, we showed that patients with high EFV had higher plasma levels of miR-15b-3p, miR-22-3p, miR-148a-3p, miR-148b-3p and miR-590-5p. Plasma miR-15b-3p, miR-22-3p, miR-148a-3p and miR-148b-3p were also directly correlated with EFV. Importantly, these miRNAs were associated with EFV even after extensive adjustment for demographic, anthropometric and clinical variables, including medication use. The results support the previously suggested link between circulating miRNAs and different fat depots [29,30,31,32,33,34]. Indeed, we have recently demonstrated that serum levels of the cardiomyocyte-enriched miR-1 and miR-133a-3p are positively correlated with myocardial steatosis in type 2 diabetes patients [35]. We then evaluated the performance of plasma miRNAs as biomarkers of EFV and EFVi. The expression of miRNAs, individually, pairwise, or in families, showed modest discrimination values for all EFV and EFVi cutoffs. The substantial overlap in plasma miRNA levels between patients with high or low levels of epicardial fat suggests that miRNAs should not be used alone to predict EFV. Conversely, the comparison of ROC curves showed that different combinations of miR-27a-3p, miR-146a-5p, miR-148b-3p and miR-152-3p improved the discrimination ability over a clinical model that included significant predictors of EFV: age, sex, BMI and diabetes mellitus. The same miRNAs correctly reclassify patients misclassified by the clinical model alone. Decision tree models supported these findings. As expected, BMI was the most powerful predictor for both EFV and EFVi in those decision trees when considering clinical variables. The inclusion of plasma miRNAs in the decision tree models yielded more specific patient subgroups. These results were observed using three independent EFV cutoffs: EFV tertiles in our study population, the EFV clinical range proposed by Spearman et al. [15] and the EFVi clinical range proposed by Shmilovich et al. [21].

According to our findings, the addition of certain miRNA signatures to clinical variables may help classify patients by their EFV. It seems that the ideal scenario for miRNA testing is based on the concept of several miRNAs-one disease contrary to the one miRNA-one disease concept, at least for EAT assessment. Therefore, the use of miRNA signatures may provide more comprehensive information for a clinical decision than the analysis of individual miRNAs. Additionally, our results suggest a potential of circulating miRNAs as biomarkers of EFV in specific patient subpopulations. These results are consistent with a new strategy that recommends the clinical application of biomarkers in individuals or subgroups of individuals as an alternative to the classical one size fits all. Overall, this study provides useful hypothesis-generating data. Using signatures of miRNAs is a promising strategy to identify biomarkers for EFV or EFVi, and subsequently, cardiometabolic disease. Because different pharmacological interventions and lifestyle changes have been proposed to treat epicardial fat accumulation [36,37], our results may have a clinical impact. The quantification of EFV is important in stratifying patients according to their cardiometabolic risk profile and evaluating the effect of treatments. However, imaging techniques are limited by methodology and operating expenses. In this context, miRNA-based tests have emerged as a cost-effective alternative for risk assessment and disease monitoring [38]. Therefore, the incorporation of a diagnostic assessment tool that combines information from clinical variables and plasma miRNA signatures into clinical workflows could provide substantial health and economic benefits in the management of cardiometabolic disease (e.g., as gatekeeper for the inclusion or exclusion of patients in subsequent imaging studies).

Epicardial fat is a source of signaling molecules that can modulate the structure and function of adjacent tissues, i.e., myocardium and vasculature. The secretion of pro-inflammatory adipocytokines from EAT into the circulatory system may also affect the systemic inflammatory state [39]. Extracellular miRNAs have been proposed to function as paracrine and endocrine signals [40,41]. Here, we have demonstrated that our miRNAs are secreted from human EAT explants and are presented in the circulation. Although the understanding of circulating ncRNA biology is still at an early stage [42], it is possible to speculate about the role of miRNAs in the cross-talk between EAT and target tissues. Indeed, miRNAs secreted from fat tissue may be novel adipokines that can regulate metabolism in distant tissues [34,43]. Previous findings are consistent with this hypothesis. miR-27a derived from adipocytes of high-fat diet mice induced insulin resistance in skeletal muscle cells, which suggests that this miRNA mediates cross-talk between adipose tissue and skeletal muscle [44]. Exosomes derived from miR-146a-modified adipose-derived stem cells attenuated myocardial damage in an acute myocardial infarction model in rats [45]. Thus, the miRNAs identified in this study may participate in regulating molecular pathways implicated in cardiometabolic physiology and pathology. Further investigation to determine the mechanisms are warranted, with a special focus on the precise role of plasma miRNAs in intercellular communication.

The strengths of this study include the use of CCTA to quantify epicardial fat, an accurate imaging method widely used in clinical studies [46], and the complete volumetric analysis of epicardial fat instead of using markers such as linear thickness. The use of plasma miRNAs as potential biomarkers was explored using three distinct classifications for epicardial fat burden, including quantitative methods proposed for standardization. In addition, decision tree models were incorporated in the evaluation of plasma miRNAs as indicators. However, the results of our study should be interpreted with respect to the study design and its limitations. First, the study population was a heterogeneous group of patients referred for CCTA. Although these patients represented a group at high cardiometabolic risk, the application of the results to other populations is limited. The results require further corroboration in a real-world setting. Nonetheless, our intention was to test the potential of using circulating miRNAs as biomarkers of epicardial fat content. Second, the classification used for the evaluation of miRNAs as biomarkers was arbitrary: EFV tertiles. As stated above, the cutoffs proposed by the bibliography have been reported as clinically relevant. However, they have not been widely accepted by clinical practice. Therefore, patients were divided into two categories: first and second EFV tertiles (low-medium epicardial fat burden) vs. third EFV tertile (high epicardial fat burden). Third, only 12 of all the candidates initially identified in the screen were further validated in the patient population. Other miRNAs may be potential biomarkers. Fourth, although EAT has unique metabolic properties and is associated with cardiovascular risk independent of other indicators of adiposity [14], it remains possible that other visceral fat depots may be confounding factors. Fifth, we cannot exclude the impact caused by physiological and pathological conditions that were not recorded in the plasma miRNA profile [47].

In any case, our results established the strength of plasma miRNAs as biomarkers for the evaluation of EFV. This investigation provides a rationale for larger and multicentric studies focused on the use of miRNAs in the routine quantification of epicardial fat burden.

## Figures and Tables

**Table 1:** Characteristics of the study population.

Variable	All	Tertile 1&2	Tertile 3	*p* Value
	*N* = 180	*N* = 120	*N* = 60	
**Clinical characteristics**				
Age (years), mean ± SD	65.0 ± 12.8	63.5 ± 13.8	68.1 ± 9.9	0.011
Male, *N* (%)	104 (58)	63 (53)	41 (68)	0.055
Body mass index (kg m^−2^), median (P25–P75)	27.0 (24.8–30.3)	25.9 (24.2–29.2)	29.4 (26.3–32.1)	<0.001
Body surface area (m^2^), median (P25–P75) *N* = 160	1.8 (1.7–2.0)	1.8 (1.7–1.9)	1.9 (1.9–2.1)	0.001
Hypertension, *N* (%)	111 (62)	70 (58)	41 (68)	0.255
Dyslipidemia, *N* (%)	102 (57)	66 (55)	36 (60)	0.632
Diabetes mellitus, *N* (%)	37 (21)	18 (15)	19 (32)	0.011
Active or former smoker, *N* (%)	59 (33)	36 (30)	23 (38)	0.310
hs-CRP (mg L^−1^), median (P25–P75)	2.00 (0.97–4.07)	1.90 (0.85–4.00)	2.10 (1.11–4.62)	0.590
Coronary artery disease, *N* (%)	55 (30.6)	35 (29.2)	20 (33.3)	0.608
Glomerular filtration rate < 60 mL/mi/1.73 m^2^, *N* (%)	16 (9)	11 (9)	5 (8)	1.000
**Medication use**				
Antiplatelet drugs, *N* (%)	73 (41)	43 (36)	40 (50)	0.071
Statins, *N* (%)	87 (48)	52 (43)	35 (58)	0.052
Beta-blockers, *N* (%)	58 (32)	34 (28)	24 (40)	0.086
Angiotensin-converting-enzyme inhibitors, *N* (%)	97 (54)	64 (53)	33 (55)	0.746
Diuretics, *N* (%)	48 (27)	31 (26)	17 (28)	0.718
**Epicardial fat burden**				
Epicardial fat volume (cm^3^), median (P25–P75)	96.0 (66.5–130.6)	79.3 (55.8–96.4)	146.4 (130.5–178.4)	<0.001
Epicardial fat volume-indexed (cm^3^ m^−2^), median (P25–P75) *N* = 160	50.0 (38.2–67.2)	42.0 (32.1–52.4)	76.3 (67.4–92.9)	<0.001

Data are presented as frequencies (percentages) for categorical variables. Continuous variables are presented as mean ± standard deviation (SD) or median (P25–P75). Differences between groups were analyzed using Student’s *t*-test, Mann–Whitney U test or Fisher’s exact test. hs-CRP: High-sensitive C-reactive protein.

**Table 2:** Association between circulating microRNAs and epicardial fat volume.

	Model 1		Model 2		Model 3		Model 4	
	OR (95% CI)	*p* Value	OR (95% CI)	*p* Value	OR (95% CI)	*p* Value	OR (95% CI)	*p* Value
**miR-15b-3p**	1.701 (1.158–2.500)	0.007	1.800 (1.177–2.753)	0.007	1.832 (1.181–2.844)	0.007	1.793 (1.174–2.738)	0.007
**miR-15b-5p**	1.132 (0.907–1.412)	0.273	1.183 (0..923–1.516)	0.185	1.168 (0.905–1.507)	0.234	1.182 (0.922–1.515)	0.188
**miR-21-5p**	1.092 (0.824–1.446)	0.540	1.169 (0.856–1.596)	0.327	1.127 (0.816–1.555)	0.468	1.168 (0.855–1.596)	0.329
**miR-22-3p**	1.551 (1.075–2.239)	0.019	1.677 (1.113–2.527)	0.013	1.655 (1.089–2.516)	0.018	1.669 (1.109–2.514)	0.014
**miR-27a-3p**	1.100 (0.865–1.398)	0.438	1.146 (0.880–1.492)	0.311	1.120 (0.852–1.471)	0.417	1.142 (0.877–1.488)	0.324
**miR-27b-3p**	1.269 (0.976–1.651)	0.076	1.331 (0.994–1.781)	0.055	1.320 (0.977–1.782)	0.070	1.325 (0.989–1.774)	0.059
**miR-146a-5p**	1.010 (0.781–1.305)	0.942	1.066 (0.805–1.410)	0.657	1.027 (0.768–1.372)	0.858	1.062 (0.801–1.406)	0.677
**miR-148a–3p**	1.387 (1.052–1.829)	0.020	1.417 (1.045–1.921)	0.025	1.429 (1.045–1.955)	0.025	1.417 (1.045–1.923)	0.025
**miR-148b-3p**	1.444 (1.081–1.929)	0.013	1.563 (1.130–2.161)	0.007	1.527 (1.096–2.128)	0.012	1.558 (1.127–2.154)	0.007
**miR-152-3p**	1.222 (0.945–1.581)	0.127	1.310 (0.982–1.748)	0.066	1.283 (0.953–1.728)	0.101	1.306 (0.979–1.744)	0.070
**miR-339-3p**	1.304 (0.985–1.726)	0.064	1.350 (0.992–1.838)	0.056	1.317 (0.960–1.806)	0.088	1.344 (0.988–1.830)	0.060
**miR-590-5p**	1.449 (1.018–2.062)	0.039	1.571 (1.062–2.324)	0.024	1.541 (1.030–2.306)	0.036	1.564 (1.059–2.312)	0.025

Model 1: Unadjusted; Model 2: Adjusted for age, sex, body mass index and diabetes mellitus; Model 3: Model 2 adjusted for antiplatelet drugs, statins use and beta-blockers use; Model 4: Model 2 adjusted for coronary artery disease. OR: odds ratio; 95% CI: 95% confidence interval.

## Supplementary Materials

The following are available online at https://www.mdpi.com/2077-0383/8/6/780/s1: Table S1: Characteristics of the study population (Screening), Table S2: Plasma microRNA screening, Table S3: microRNA candidates, Table S4: Correlations between epicardial fat volume and plasma microRNAs, Figure S1: Calibration plots (with internal validation), Figure S2: Plasma microRNAs as biomarkers of epicardial fat volume-indexed (EFVi), according to the cutoff proposed by Shmilovich et al.

## References

[1] Mendell J.T., Olson E.N. (2012). MicroRNAs in stress signaling and human disease. Cell.

[2] Mitchell P.S., Parkin R.K., Kroh E.M., Fritz B.R., Wyman S.K., Pogosova-Agadjanyan E.L., Peterson A., Noteboom J., O’Briant K.C., Allen A. (2008). Circulating microRNAs as stable blood-based markers for cancer detection. Proc. Natl. Acad. Sci. USA.

[3] De Gonzalo-Calvo D., Vea A., Bar C., Fiedler J., Couch L.S., Brotons C., Llorente-Cortes V., Thum T. (2019). Circulating non-coding RNAs in biomarker-guided cardiovascular therapy: A novel tool for personalized medicine?. Eur. Heart J..

[4] Bianchi F., Nicassio F., Marzi M., Belloni E., Dall’olio V., Bernard L., Pelosi G., Maisonneuve P., Veronesi G., Di Fiore P.P. (2011). A serum circulating miRNA diagnostic test to identify asymptomatic high-risk individuals with early stage lung cancer. EMBO Mol. Med..

[5] Ralfkiaer U., Hagedorn P.H., Bangsgaard N., Lovendorf M.B., Ahler C.B., Svensson L., Kopp K.L., Vennegaard M.T., Lauenborg B., Zibert J.R. (2011). Diagnostic microRNA profiling in cutaneous T-cell lymphoma (CTCL). Blood.

[6] De Gonzalo-Calvo D., Iglesias-Gutierrez E., Llorente-Cortes V. (2017). Epigenetic Biomarkers and Cardiovascular Disease: Circulating MicroRNAs. Rev. Esp. Cardiol..

[7] Guay C., Regazzi R. (2013). Circulating microRNAs as novel biomarkers for diabetes mellitus. Nat. Rev. Endocrinol..

[8] Iacobellis G. (2015). Local and systemic effects of the multifaceted epicardial adipose tissue depot. Nat. Rev. Endocrinol..

[9] Packer M. (2018). The epicardial adipose inflammatory triad: Coronary atherosclerosis, atrial fibrillation, and heart failure with a preserved ejection fraction. Eur. J. Heart Fail..

[10] Ansaldo A.M., Montecucco F., Sahebkar A., Dallegri F., Carbone F. (2019). Epicardial adipose tissue and cardiovascular diseases. Int. J. Cardiol..

[11] Blumensatt M., Fahlbusch P., Hilgers R., Bekaert M., Herzfeld de Wiza D., Akhyari P., Ruige J.B., Ouwens D.M. (2017). Secretory products from epicardial adipose tissue from patients with type 2 diabetes impair mitochondrial beta-oxidation in cardiomyocytes via activation of the cardiac renin-angiotensin system and induction of miR-208a. Basic Res. Cardiol..

[12] Mancio J., Azevedo D., Saraiva F., Azevedo A.I., Pires-Morais G., Leite-Moreira A., Falcao-Pires I., Lunet N., Bettencourt N. (2018). Epicardial adipose tissue volume assessed by computed tomography and coronary artery disease: A systematic review and meta-analysis. Eur. Heart J. Cardiovasc. Imaging.

[13] Nyman K., Graner M., Pentikainen M.O., Lundbom J., Hakkarainen A., Siren R., Nieminen M.S., Taskinen M.R., Lundbom N., Lauerma K. (2013). Cardiac steatosis and left ventricular function in men with metabolic syndrome. J. Cardiovasc. Magn. Reson..

[14] Bos D., Vernooij M.W., Shahzad R., Kavousi M., Hofman A., van Walsum T., Deckers J.W., Ikram M.A., Heeringa J., Franco O.H. (2017). Epicardial Fat Volume and the Risk of Atrial Fibrillation in the General Population Free of Cardiovascular Disease. JACC Cardiovasc. Imaging.

[15] Spearman J.V., Renker M., Schoepf U.J., Krazinski A.W., Herbert T.L., De Cecco C.N., Nietert P.J., Meinel F.G. (2015). Prognostic value of epicardial fat volume measurements by computed tomography: A systematic review of the literature. Eur. Radiol..

[16] Pierdomenico S.D., Pierdomenico A.M., Cuccurullo F., Iacobellis G. (2013). Meta-analysis of the relation of echocardiographic epicardial adipose tissue thickness and the metabolic syndrome. Am. J. Cardiol..

[17] Beltowski J. (2019). Epicardial adipose tissue: The new target for statin therapy. Int. J. Cardiol..

[18] Iacobellis G., Mohseni M., Bianco S.D., Banga P.K. (2017). Liraglutide causes large and rapid epicardial fat reduction. Obesity.

[19] De Gonzalo-Calvo D., Vilades D., Nasarre L., Carreras F., Leta R., Garcia-Moll X., Llorente-Cortes V. (2016). Circulating levels of soluble low-density lipoprotein receptor-related protein 1 (sLRP1) as novel biomarker of epicardial adipose tissue. Int. J. Cardiol..

[20] Gonzalo-Calvo D., Colom C., Vilades D., Rivas-Urbina A., Moustafa A.H., Perez-Cuellar M., Sanchez-Quesada J.L., Perez A., Llorente-Cortes V. (2018). Soluble LRP1 is an independent biomarker of epicardial fat volume in patients with type 1 diabetes mellitus. Sci. Rep..

[21] Shmilovich H., Dey D., Cheng V.Y., Rajani R., Nakazato R., Otaki Y., Nakanishi R., Slomka P.J., Thomson L.E., Hayes S.W. (2011). Threshold for the upper normal limit of indexed epicardial fat volume: Derivation in a healthy population and validation in an outcome-based study. Am. J. Cardiol..

[22] Tuck M.K., Chan D.W., Chia D., Godwin A.K., Grizzle W.E., Krueger K.E., Rom W., Sanda M., Sorbara L., Stass S. (2009). Standard operating procedures for serum and plasma collection: early detection research network consensus statement standard operating procedure integration working group. J. Proteome Res..

[23] Mestdagh P., Hartmann N., Baeriswyl L., Andreasen D., Bernard N., Chen C., Cheo D., D’Andrade P., DeMayo M., Dennis L. (2014). Evaluation of quantitative miRNA expression platforms in the microRNA quality control (miRQC) study. Nat. Methods.

[24] Metsalu T., Vilo J. (2015). ClustVis: A web tool for visualizing clustering of multivariate data using Principal Component Analysis and heatmap. Nucleic Acids Res..

[25] DeLong E.R., DeLong D.M., Clarke-Pearson D.L. (1988). Comparing the areas under two or more correlated receiver operating characteristic curves: A nonparametric approach. Biometrics.

[26] Pencina M.J., D’Agostino R.B., Steyerberg E.W. (2011). Extensions of net reclassification improvement calculations to measure usefulness of new biomarkers. Stat. Med..

[27] Harrell F.E. (2015). Regression Modeling Strategies. Springer Series in Statistics.

[28] Kass G. (1980). An exploratory technique for investigating large quantities of categorical data. Appl. Stat..

[29] Chen Y., Buyel J.J., Hanssen M.J., Siegel F., Pan R., Naumann J., Schell M., van der Lans A., Schlein C., Froehlich H. (2016). Exosomal microRNA miR-92a concentration in serum reflects human brown fat activity. Nat. Commun..

[30] Cui X., You L., Zhu L., Wang X., Zhou Y., Li Y., Wen J., Xia Y., Wang X., Ji C. (2018). Change in circulating microRNA profile of obese children indicates future risk of adult diabetes. Metabolism.

[31] Heneghan H.M., Miller N., McAnena O.J., O’Brien T., Kerin M.J. (2011). Differential miRNA expression in omental adipose tissue and in the circulation of obese patients identifies novel metabolic biomarkers. J. Clin. Endocrinol. Metab..

[32] Pek S.L., Sum C.F., Lin M.X., Cheng A.K., Wong M.T., Lim S.C., Tavintharan S. (2016). Circulating and visceral adipose miR-100 is down-regulated in patients with obesity and Type 2 diabetes. Mol. Cell Endocrinol..

[33] Prats-Puig A., Ortega F.J., Mercader J.M., Moreno-Navarrete J.M., Moreno M., Bonet N., Ricart W., Lopez-Bermejo A., Fernandez-Real J.M. (2013). Changes in circulating microRNAs are associated with childhood obesity. J. Clin. Endocrinol. Metab..

[34] Thomou T., Mori M.A., Dreyfuss J.M., Konishi M., Sakaguchi M., Wolfrum C., Rao T.N., Winnay J.N., Garcia-Martin R., Grinspoon S.K. (2017). Adipose-derived circulating miRNAs regulate gene expression in other tissues. Nature.

[35] De Gonzalo-Calvo D., van der Meer R.W., Rijzewijk L.J., Smit J.W., Revuelta-Lopez E., Nasarre L., Escola-Gil J.C., Lamb H.J., Llorente-Cortes V. (2017). Serum microRNA-1 and microRNA-133a levels reflect myocardial steatosis in uncomplicated type 2 diabetes. Sci. Rep..

[36] Nakazato R., Rajani R., Cheng V.Y., Shmilovich H., Nakanishi R., Otaki Y., Gransar H., Slomka P.J., Hayes S.W., Thomson L.E. (2012). Weight change modulates epicardial fat burden: A 4-year serial study with non-contrast computed tomography. Atherosclerosis.

[37] Parisi V., Petraglia L., D’Esposito V., Cabaro S., Rengo G., Caruso A., Grimaldi M.G., Baldascino F., De Bellis A., Vitale D. (2019). Statin therapy modulates thickness and inflammatory profile of human epicardial adipose tissue. Int. J. Cardiol..

[38] Walter E., Dellago H., Grillari J., Dimai H.P., Hackl M. (2018). Cost-utility analysis of fracture risk assessment using microRNAs compared with standard tools and no monitoring in the Austrian female population. Bone.

[39] Packer M. (2018). Epicardial Adipose Tissue May Mediate Deleterious Effects of Obesity and Inflammation on the Myocardium. J. Am. Coll. Cardiol..

[40] Bang C., Batkai S., Dangwal S., Gupta S.K., Foinquinos A., Holzmann A., Just A., Remke J., Zimmer K., Zeug A. (2014). Cardiac fibroblast-derived microRNA passenger strand-enriched exosomes mediate cardiomyocyte hypertrophy. J. Clin. Invest..

[41] Shan Z., Qin S., Li W., Wu W., Yang J., Chu M., Li X., Huo Y., Schaer G.L., Wang S. (2015). An Endocrine Genetic Signal Between Blood Cells and Vascular Smooth Muscle Cells: Role of MicroRNA-223 in Smooth Muscle Function and Atherogenesis. J. Am. Coll. Cardiol..

[42] Bär C., Thum T., de Gonzalo-Calvo D. (2019). Circulating miRNAs as mediators in cell-to-cell communication. Epigenomics.

[43] Ying W., Riopel M., Bandyopadhyay G., Dong Y., Birmingham A., Seo J.B., Ofrecio J.M., Wollam J., Hernandez-Carretero A., Fu W. (2017). Adipose Tissue Macrophage-Derived Exosomal miRNAs Can Modulate In Vivo and In Vitro Insulin Sensitivity. Cell.

[44] Yu Y., Du H., Wei S., Feng L., Li J., Yao F., Zhang M., Hatch G.M., Chen L. (2018). Adipocyte-Derived Exosomal MiR-27a Induces Insulin Resistance in Skeletal Muscle Through Repression of PPARgamma. Theranostics.

[45] Pan J., Alimujiang M., Chen Q., Shi H., Luo X. (2018). Exosomes derived from miR-146a-modified adipose-derived stem cells attenuate acute myocardial infarction-induced myocardial damage via downregulation of early growth response factor 1. J. Cell. Biochem..

[46] Raggi P. (2013). Epicardial adipose tissue as a marker of coronary artery disease risk. J. Am. Coll. Cardiol..

[47] De Gonzalo-Calvo D., Davalos A., Fernandez-Sanjurjo M., Amado-Rodriguez L., Diaz-Coto S., Tomas-Zapico C., Montero A., Garcia-Gonzalez A., Llorente-Cortes V., Heras M.E. (2018). Circulating microRNAs as emerging cardiac biomarkers responsive to acute exercise. Int. J. Cardiol..

