# Supplementary material for: Plasma microRNA Profiling Reveals Novel Biomarkers of Epicardial Adipose Tissue: A Multidetector Computed Tomography Study
Source: J Clin Med. 2019 Jun 1;8(6):780. doi: 10.3390/jcm8060780 (PMC6616954; doi:10.3390/jcm8060780)
Supplement: Supplementary file 1 [file jcm-08-00780-s001.pdf]

Supplemental Table S1. Characteristics of the study population (Screening).

Variable	Tertile 1<br>N = 8	Tertile 3<br>N = 8	P-value
Age (years), mean ± SD	67.3 ± 9.3	66.8 ± 9.0	0.914
Male, N (%)	3 (38)	3 (38)	1.000
Body mass index (kg/m^2), median (P25-P75)	26.3 (24.7-28.5)	26.0 (24.1-27.6)	0.959
Hypertension, N (%)	3 (38)	4 (50)	1.000
Dyslipidemia, N (%)	7 (88)	5 (63)	0.569
Diabetes mellitus, N (%)	0 (0)	0 (0)	1.000
Active or former smoker, N (%)	1 (13)	1 (13)	1.000
hs-CRP (mg/L), median (P25-P75)	1.22 (0.98-2.61)	2.16 (0.74-3.08)	0.590
Coronary artery disease, N (%)	1 (13)	1 (13)	1.000
Epicardial fat volume (cm^3), median (P25-P75)	43.6 (24.7-60.4)	139.8 (120.9-165.1)	<0.001

Data are presented as frequencies (percentages) for categorical variables. Continuous variables are presented as mean ± standard deviation (SD) or median (P25-P75). Differences between groups were analyzed using Student´s t test, Mann-Whitney U test or Fisher's exact test.

Supplemental Table S2. Plasma microRNA screening.

microRNA	Tertile 1		Tertile 3		P-value	Median Cq	Min Cq	Max Cq
hsa-let-7a-5p	14.85	(14.41-15.07)	15.71	(14.99-16.58)	0.105	25.2	23.4	26.3
hsa-let-7b-3p	8.17	(8.1-8.93)	8.87	(8.37-9.56)	0.105	31.9	30.2	32.5
hsa-let-7b-5p	14.54	(14-14.92)	15.48	(15.04-16.37)	0.050	25.2	24.1	26.8
hsa-let-7c-5p	10.58	(9.65-10.83)	11.31	(10.26-12.53)	0.161	29.5	27.7	30.8
hsa-let-7d-3p	13.49	(13.25-13.94)	14.29	(13.76-15.28)	0.050	26.5	24.8	27.3
hsa-let-7d-5p	11.86	(11.31-12.05)	12.78	(11.7-13.92)	0.105	28.2	25.8	29.6
hsa-let-7e-5p	10.00	(9.21-10.72)	11.17	(10.03-12.12)	0.161	30.0	28.0	31.4
hsa-let-7f-5p	12.99	(12.41-13.32)	14.02	(13.33-15.18)	0.050	26.9	24.7	28.2
hsa-let-7g-5p	14.40	(13.55-14.69)	15.28	(14.57-16.38)	0.065	25.7	23.6	26.9
hsa-let-7i-5p	13.21	(13.14-13.57)	13.89	(13.83-14.59)	0.050	26.8	25.5	27.6
hsa-miR-1	8.07	(7.13-9)	9.48	(7.74-10.48)	0.279	31.8	28.4	34.9
hsa-miR-100-5p	8.58	(7.35-9.29)	8.83	(8.55-9.12)	0.574	31.8	30.3	33.5
hsa-miR-101-3p	14.74	(14.15-15.06)	15.26	(14.96-15.88)	0.083	25.4	23.6	26.3
hsa-miR-103a-3p	15.31	(14.41-15.43)	16.19	(15.2-17.46)	0.105	24.9	22.3	26.4
hsa-miR-106a-5p	16.57	(15.87-16.79)	17.26	(16.99-18.13)	0.021	23.5	21.9	24.4
hsa-miR-106b-3p	9.31	(9.19-9.6)	10.47	(9.44-11.28)	0.083	30.9	29.0	32.2
hsa-miR-106b-5p	13.06	(12.64-13.55)	14.14	(13.21-14.69)	0.195	27.0	25.4	28.2
hsa-miR-107	12.89	(12.29-13.2)	13.75	(12.92-15.1)	0.105	27.2	24.8	28.3
hsa-miR-10b-5p	10.81	(9.81-11.14)	10.97	(10.39-11.62)	0.442	29.5	28.1	30.9
hsa-miR-122-5p	12.83	(12.18-13.91)	13.57	(11.88-14.99)	0.574	27.2	22.7	30.1
hsa-miR-125a-5p	11.41	(10.6-12.19)	12.26	(11.34-13.72)	0.234	28.7	26.5	30.4
hsa-miR-125b-5p	11.91	(10.3-12.52)	12.77	(12.09-13.37)	0.105	28.2	26.6	30.4
hsa-miR-1260a	10.04	(9.73-10.57)	10.54	(10.3-11.02)	0.130	30.1	29.2	31.3
hsa-miR-126-3p	17.13	(16.44-17.34)	18.03	(17.19-19.25)	0.065	22.9	21.1	24.0
hsa-miR-126-5p	13.53	(13.03-13.96)	14.30	(13.98-15.59)	0.021	26.3	24.5	27.4
hsa-miR-127-3p	8.81	(7.34-9.56)	9.58	(7.42-10.63)	0.442	31.4	28.2	34.2
hsa-miR-128-3p	9.92	(9.75-10.04)	10.42	(9.91-11.25)	0.195	30.3	28.5	30.8
hsa-miR-130a-3p	11.19	(10.67-11.59)	11.98	(11.8-12.95)	0.015	28.7	27.0	29.9
hsa-miR-130b-3p	8.66	(8.53-8.95)	9.49	(9.19-10.35)	0.015	31.2	29.3	32.2
hsa-miR-132-3p	10.01	(9.54-10.2)	10.48	(10.09-11.16)	0.083	30.1	28.8	31.3
hsa-miR-133a-3p	8.44	(8.21-9.36)	9.49	(8.13-10.89)	0.279	31.6	28.3	34.5
hsa-miR-133b	9.24	(7.95-9.85)	10.35	(9.57-11.82)	0.083	30.7	27.8	33.9
hsa-miR-136-3p	7.95	(7.04-9.63)	8.62	(6.63-10.38)	0.955	32.6	22.6	34.0
hsa-miR-136-5p	7.36	(7.03-7.83)	6.86	(5.29-8.97)	0.721	33.1	29.9	35.0
hsa-miR-139-5p	11.17	(10.48-11.94)	12.18	(10.07-13.27)	0.442	28.9	27.1	30.7
hsa-miR-140-3p	14.27	(13.6-14.57)	14.60	(13.9-15.27)	0.328	26.0	24.7	27.0
hsa-miR-140-5p	11.46	(10.99-11.77)	12.14	(11.84-13.32)	0.021	28.7	26.9	29.8
hsa-miR-141-3p	7.68	(6.8-8.31)	8.24	(7.81-9.25)	0.279	32.2	30.9	35.0
hsa-miR-142-3p	15.30	(14.57-15.57)	16.19	(15.22-16.99)	0.161	24.9	22.5	26.4
hsa-miR-142-5p	12.09	(11.94-12.35)	13.05	(12.53-14.02)	0.015	27.9	25.5	28.7
hsa-miR-143-3p	12.46	(10.87-13.17)	12.67	(12.28-13.7)	0.574	27.6	25.1	29.4
hsa-miR-144-3p	13.50	(12.96-14.07)	13.61	(13.21-14.12)	0.798	26.9	25.2	27.8
hsa-miR-144-5p	10.03	(9.4-10.64)	10.19	(10-10.28)	0.645	30.3	29.2	31.1
hsa-miR-145-5p	11.54	(10.85-12.41)	12.51	(11.84-13.97)	0.083	28.0	25.8	29.7

**hsa-miR-146a-5p**	**14.08**	**(13.71-14.44)**	**15.08**	**(14.65-16.11)**	**0.010**	**25.7**	**24.0**	**26.7**
hsa-miR-146b-5p	8.40	(7.37-9.16)	9.18	(8.9-10.64)	0.083	31.3	29.6	33.1
**hsa-miR-148a-3p**	**12.11**	**(11.86-12.57)**	**12.72**	**(12.54-13.16)**	**0.065**	**27.9**	**26.1**	**29.1**
**hsa-miR-148b-3p**	**13.09**	**(12.9-13.37)**	**13.91**	**(13.65-14.64)**	**0.010**	**26.8**	**24.4**	**27.5**
hsa-miR-150-5p	14.04	(13.29-14.19)	14.10	(13.78-14.86)	0.505	26.2	25.5	27.3
hsa-miR-151a-3p	11.91	(11.46-12.26)	12.85	(12.37-14.14)	0.015	27.9	26.1	29.1
hsa-miR-151a-5p	13.98	(13.69-14.55)	15.01	(14.27-16.08)	0.038	25.8	24.0	26.9
**hsa-miR-152-3p**	**11.58**	**(10.49-12)**	**12.59**	**(12.03-12.96)**	**0.028**	**28.3**	**26.7**	**29.9**
hsa-miR-154-5p	10.39	(9.66-10.94)	10.57	(8.9-11.71)	0.798	30.0	27.7	33.3
hsa-miR-155-5p	8.54	(7.92-9.1)	9.60	(8.68-10.73)	0.105	31.2	29.3	33.4
hsa-miR-15a-5p	16.02	(15.78-16.52)	16.31	(16.21-17.26)	0.083	24.2	22.8	24.7
**hsa-miR-15b-3p**	**11.67**	**(11.54-11.72)**	**12.31**	**(11.92-12.98)**	**0.010**	**28.5**	**27.2**	**29.1**
**hsa-miR-15b-5p**	**12.36**	**(12.12-12.67)**	**13.25**	**(13.06-14.55)**	**0.007**	**27.4**	**25.4**	**28.6**
hsa-miR-16-2-3p	10.77	(10.17-11.41)	11.10	(10.65-11.59)	0.382	29.5	28.4	30.3
hsa-miR-16-5p	21.25	(20.48-21.52)	21.57	(21.09-22.16)	0.130	18.9	17.8	19.9
hsa-miR-17-5p	10.05	(9.36-10.37)	10.81	(10.51-11.74)	0.021	29.8	28.5	31.0
hsa-miR-181a-5p	11.69	(11.02-12.67)	12.96	(12.07-13.64)	0.234	27.9	26.1	29.6
hsa-miR-185-5p	16.67	(16.51-17.03)	17.35	(17.12-18.22)	0.038	23.4	22.2	24.1
hsa-miR-186-5p	8.98	(8.59-9.54)	9.92	(9.47-10.7)	0.050	30.9	29.4	32.3
hsa-miR-18a-5p	13.28	(12.55-13.5)	13.90	(13.77-15.71)	0.040	26.8	24.8	28.2
hsa-miR-18b-5p	13.01	(12.21-13.25)	13.66	(13.34-14.95)	0.028	27.0	25.2	28.4
hsa-miR-191-5p	14.62	(13.74-15.06)	15.42	(14.95-17.09)	0.038	25.3	23.3	26.8
hsa-miR-192-5p	11.92	(10.99-12.74)	12.35	(11.73-12.8)	0.574	28.3	25.7	29.5
hsa-miR-193a-5p	7.31	(5.92-7.75)	7.28	(7.01-7.83)	0.721	33.1	31.4	34.9
hsa-miR-194-5p	10.01	(9.27-10.56)	10.49	(10.04-10.98)	0.279	30.1	28.1	31.4
hsa-miR-195-5p	8.67	(8.55-9.72)	9.88	(8.75-10.21)	0.195	30.9	29.7	32.5
hsa-miR-197-3p	12.07	(11.52-12.46)	12.75	(12.14-13.89)	0.083	27.8	26.3	29.2
hsa-miR-199a-3p	13.54	(13.01-13.95)	14.43	(13.78-15.17)	0.083	26.5	24.2	27.3
hsa-miR-199a-5p	11.27	(10.52-11.52)	12.27	(11.2-13.18)	0.161	28.8	26.2	30.0
hsa-miR-19a-3p	16.89	(16.62-17.23)	17.46	(17.13-18.14)	0.083	23.3	21.5	24.0
hsa-miR-19b-3p	18.10	(17.7-18.58)	18.74	(18.37-19.36)	0.083	22.1	20.6	22.7
hsa-miR-200a-3p	5.31	(5.06-5.82)	6.33	(5.85-7.21)	0.015	34.6	32.2	35.0
hsa-miR-200c-3p	7.88	(7.32-8.38)	8.87	(7.9-10.15)	0.083	32.0	30.1	33.1
hsa-miR-205-5p	6.07	(5.21-6.72)	6.13	(5.23-6.96)	0.798	34.0	32.8	35.0
hsa-miR-20a-5p	16.66	(16.17-16.97)	17.43	(17.13-18.26)	0.021	23.3	22.0	24.2
hsa-miR-20b-5p	7.56	(7.33-8.15)	8.17	(7.36-8.35)	0.382	32.5	31.6	34.1
hsa-miR-210-3p	10.35	(9.83-10.8)	11.33	(10.68-11.41)	0.065	29.7	28.7	30.5
hsa-miR-2110	7.81	(7.27-8.19)	8.84	(8.48-9.46)	0.028	32.1	31.1	33.3
hsa-miR-215-5p	10.67	(9.78-11.5)	11.30	(10.52-11.81)	0.279	29.4	26.6	31.2
**hsa-miR-21-5p**	**17.16**	**(16.66-17.58)**	**17.87**	**(17.68-18.77)**	**0.015**	**22.7**	**21.0**	**24.9**
hsa-miR-221-3p	15.55	(15.34-15.76)	16.49	(15.83-17.45)	0.038	24.4	22.6	25.7
hsa-miR-222-3p	13.72	(13.26-14.06)	14.35	(14.18-14.9)	0.038	26.2	25.3	27.3
hsa-miR-223-3p	19.39	(18.83-19.54)	20.23	(19.52-21.45)	0.050	20.7	18.8	22.0
hsa-miR-223-5p	8.73	(8.49-9.04)	9.43	(8.62-10.57)	0.161	31.3	29.6	32.1
**hsa-miR-22-3p**	**13.46**	**(13.18-13.5)**	**14.07**	**(13.73-14.58)**	**0.010**	**26.7**	**25.0**	**27.4**
hsa-miR-22-5p	9.39	(9.04-10.02)	9.75	(9.44-10.55)	0.442	30.8	29.1	32.0
hsa-miR-23a-3p	17.41	(16.7-17.66)	18.21	(17.68-19.42)	0.050	22.6	20.7	23.6
hsa-miR-23b-3p	15.18	(14.62-15.51)	16.18	(15.58-17.5)	0.028	24.7	22.7	25.8

hsa-miR-24-3p	16.96	(16.73-17.44)	18.09	(17.45-18.84)	0.021	22.8	21.3	23.8
hsa-miR-25-3p	16.44	(16-16.93)	16.78	(16.58-17.69)	0.105	23.8	22.3	24.5
hsa-miR-26a-5p	13.12	(12.18-13.67)	14.18	(13.13-15.79)	0.105	26.8	24.4	28.3
hsa-miR-26b-5p	11.67	(10.87-12.32)	12.79	(12.42-14.26)	0.050	27.9	25.8	29.9
**hsa-miR-27a-3p**	**13.38**	**(12.97-13.73)**	**14.36**	**(14-15.59)**	**0.028**	**26.4**	**23.9**	**27.5**
**hsa-miR-27b-3p**	**15.23**	**(14.57-15.48)**	**16.08**	**(15.73-17.25)**	**0.010**	**24.6**	**22.6**	**25.7**
hsa-miR-28-3p	10.91	(9.76-11.04)	11.64	(11.21-12.96)	0.028	29.1	27.3	31.0
hsa-miR-28-5p	10.98	(9.74-11.22)	11.93	(11.15-13.17)	0.083	29.1	26.8	30.9
hsa-miR-29a-3p	11.47	(10.83-11.96)	11.67	(11.44-12.32)	0.505	28.7	27.6	30.0
hsa-miR-29b-3p	10.62	(10.06-10.91)	11.24	(10.65-11.77)	0.234	29.5	27.4	30.4
hsa-miR-29c-3p	11.80	(11-11.92)	12.24	(12.05-12.87)	0.050	28.4	26.8	29.5
hsa-miR-301a-3p	11.19	(10.7-11.62)	12.33	(11.79-13.74)	0.028	28.5	26.5	30.8
hsa-miR-30a-5p	10.13	(9.34-10.57)	10.78	(10.62-11.29)	0.065	29.9	29.0	31.3
hsa-miR-30b-5p	13.37	(12.57-13.94)	14.30	(13.24-15.65)	0.065	26.6	24.1	28.2
hsa-miR-30c-5p	13.03	(12.5-13.54)	14.01	(13.27-14.92)	0.065	26.9	25.1	27.8
hsa-miR-30d-5p	15.21	(14.68-15.5)	16.10	(15.82-17.33)	0.015	24.6	23.0	25.8
hsa-miR-30e-3p	8.66	(7.8-9.14)	9.42	(9.08-10.78)	0.065	31.1	29.4	32.7
hsa-miR-30e-5p	14.74	(14.45-15.08)	15.42	(15.06-16.44)	0.038	25.3	23.9	26.1
hsa-miR-320a	15.54	(15.05-15.83)	16.00	(15.73-16.52)	0.050	24.6	23.8	25.6
hsa-miR-320b	13.24	(12.76-13.6)	13.95	(13.65-14.38)	0.015	26.9	25.7	27.7
hsa-miR-320c	12.26	(11.83-12.55)	12.75	(12.4-13.28)	0.028	27.9	26.8	28.6
hsa-miR-320d	11.02	(10.54-11.47)	11.35	(11.09-12.04)	0.161	29.2	28.2	30.0
hsa-miR-324-3p	10.82	(10.25-11.17)	11.64	(11.46-12.1)	0.083	29.2	27.8	30.6
hsa-miR-324-5p	11.54	(10.81-11.61)	12.37	(11.8-13.28)	0.038	28.5	26.7	30.1
hsa-miR-32-5p	9.44	(9.09-9.87)	9.79	(9.6-10.01)	0.382	30.8	28.8	31.7
hsa-miR-326	8.00	(7.25-9.03)	8.83	(8.39-10.16)	0.130	31.9	28.9	33.2
hsa-miR-328-3p	11.71	(11.12-12.12)	12.82	(12.03-13.81)	0.038	28.2	26.4	29.4
hsa-miR-331-3p	10.56	(9.96-10.98)	11.76	(10.9-13.03)	0.050	29.3	27.2	30.5
hsa-miR-335-3p	9.02	(8.55-9.47)	10.15	(8.92-10.63)	0.195	31.0	28.3	32.6
hsa-miR-335-5p	8.51	(7.9-8.84)	9.60	(8.91-10.5)	0.021	31.4	28.7	33.0
hsa-miR-338-3p	10.16	(9.71-10.59)	11.19	(10.36-12.24)	0.050	29.9	27.7	31.2
**hsa-miR-339-3p**	**10.04**	**(9.64-10.08)**	**10.71**	**(10.38-11.96)**	**0.007**	**30.0**	**28.1**	**30.9**
hsa-miR-339-5p	11.39	(10.61-11.63)	12.05	(11.5-13.72)	0.050	28.7	26.3	29.7
hsa-miR-33a-5p	7.51	(7.14-8.77)	8.48	(7.44-9.55)	0.234	32.3	29.3	34.5
hsa-miR-342-3p	11.68	(10.83-12.13)	11.80	(11.1-12.8)	0.645	28.7	27.6	30.3
hsa-miR-34a-5p	9.00	(8.57-9.26)	8.76	(7.96-9.44)	0.574	31.5	30.6	33.3
hsa-miR-361-5p	12.79	(12.56-13.27)	13.56	(13.32-14.68)	0.021	27.0	25.6	27.9
hsa-miR-362-3p	8.48	(8.04-8.71)	9.17	(8.79-9.55)	0.050	31.7	30.0	32.4
hsa-miR-363-3p	12.26	(11.8-12.58)	12.30	(11.93-12.72)	0.798	28.1	27.3	29.3
hsa-miR-365a-3p	7.62	(6.36-8.66)	8.29	(7.09-9.07)	0.574	32.4	30.0	35.0
hsa-miR-374a-5p	9.40	(8.8-10.1)	10.54	(9.76-12.24)	0.038	30.3	27.8	31.7
hsa-miR-374b-5p	11.41	(10.5-11.83)	12.37	(11.58-14.02)	0.083	28.5	25.9	30.5
hsa-miR-375	8.47	(7.87-8.72)	9.03	(8.7-10.19)	0.021	31.7	29.9	34.6
hsa-miR-376a-3p	9.92	(9.39-10.08)	10.00	(8.41-11.2)	0.798	30.4	27.7	33.7
hsa-miR-376c-3p	11.89	(11.26-12.12)	12.03	(10.34-13.31)	0.645	28.4	25.8	32.5
hsa-miR-382-5p	10.87	(10.23-11.09)	11.29	(9.76-12.58)	0.234	29.3	27.1	32.1
hsa-miR-409-3p	11.06	(10.19-11.37)	11.47	(9.35-12.72)	0.382	29.3	26.6	32.8
hsa-miR-421	9.39	(8.94-10.01)	10.35	(10-11.55)	0.028	30.4	28.5	31.7

hsa-miR-423-3p	12.65	(12.08-12.98)	13.61	(12.97-14.7)	0.050	27.3	25.3	28.4
hsa-miR-423-5p	13.33	(13.12-13.77)	13.78	(13.7-14.6)	0.083	26.7	25.6	27.2
hsa-miR-424-5p	11.04	(10.39-11.46)	11.03	(9.87-11.63)	0.878	29.4	27.5	31.4
hsa-miR-425-3p	10.49	(10.06-10.82)	11.34	(10.93-12.48)	0.021	29.3	27.6	30.4
hsa-miR-425-5p	14.02	(13.61-14.48)	14.62	(14.43-15.37)	0.105	26.2	24.7	26.8
hsa-miR-451a	21.29	(20.38-21.64)	21.24	(20.72-21.77)	0.645	19.2	17.7	20.5
hsa-miR-454-3p	8.89	(8.19-9.28)	10.07	(9.44-11.17)	0.015	30.8	29.2	32.8
hsa-miR-483-5p	7.00	(6.75-7.49)	7.54	(7.08-7.95)	0.234	33.0	31.7	35.0
hsa-miR-484	12.69	(12.33-13)	13.32	(13.25-14.37)	0.028	27.4	25.7	28.0
hsa-miR-485-3p	9.44	(8.81-10.06)	10.01	(7.13-10.9)	0.721	31.0	28.1	34.4
hsa-miR-486-5p	16.65	(16.12-17.04)	16.94	(16.35-17.49)	0.382	23.6	22.2	24.3
hsa-miR-495-3p	9.57	(9.05-10.32)	9.59	(7.74-10.74)	0.959	30.6	28.2	33.4
hsa-miR-497-5p	7.75	(7.4-8.23)	7.63	(7.26-7.74)	0.505	32.7	30.4	33.3
hsa-miR-501-3p	8.20	(7.95-8.93)	8.63	(8.45-8.94)	0.083	31.8	31.1	32.8
hsa-miR-502-3p	9.09	(8.66-9.69)	9.52	(9.13-9.9)	0.195	30.9	30.4	31.9
hsa-miR-505-3p	9.79	(9.27-10.05)	10.50	(10.17-11.24)	0.038	30.1	28.8	31.3
hsa-miR-532-3p	9.26	(9.1-9.73)	9.86	(9.46-10.57)	0.161	30.7	29.7	31.9
hsa-miR-532-5p	10.43	(9.76-10.94)	11.00	(10.66-11.4)	0.083	29.6	28.6	30.7
hsa-miR-543	9.68	(8.73-9.93)	10.09	(8.35-10.86)	0.442	30.6	28.4	34.4
hsa-miR-574-3p	10.18	(9.33-10.71)	10.78	(10.34-12.26)	0.105	29.8	27.9	31.3
hsa-miR-584-5p	8.98	(8.3-9.27)	10.02	(9.59-10.92)	0.038	30.9	28.9	32.0
**hsa-miR-590-5p**	**11.81**	**(11.63-11.91)**	**12.64**	**(12.4-13.61)**	**0.010**	**28.1**	**25.9**	**29.0**
hsa-miR-629-5p	8.79	(8.27-9.24)	9.08	(8.56-9.33)	0.328	31.3	30.7	32.2
hsa-miR-652-3p	13.01	(12.28-13.16)	13.84	(13.19-14.91)	0.038	27.1	25.3	28.3
hsa-miR-660-5p	12.48	(12.02-12.7)	12.95	(12.61-13.18)	0.130	27.7	26.9	28.9
hsa-miR-7-1-3p	8.91	(8.33-9.11)	9.76	(9.31-10.73)	0.050	31.2	29.0	32.2
hsa-miR-7-5p	8.38	(7.24-9.08)	8.56	(7.59-9.29)	0.505	31.9	30.1	33.5
hsa-miR-766-3p	10.10	(9.48-10.52)	11.18	(10.41-12.82)	0.015	29.8	27.2	31.7
hsa-miR-874-3p	7.06	(6.08-7.38)	7.59	(7.07-8.27)	0.161	33.1	31.9	35.0
hsa-miR-877-5p	7.97	(7.27-8.64)	8.70	(8.54-9.5)	0.105	32.0	30.2	33.7
hsa-miR-885-5p	7.27	(5.37-8.83)	8.12	(6.86-9.09)	0.279	32.9	29.4	35.0
hsa-miR-92a-3p	16.66	(16.2-17.03)	17.25	(16.99-17.74)	0.021	23.4	22.4	24.1
hsa-miR-92b-3p	6.58	(5.87-6.98)	7.47	(6.9-8.16)	0.028	33.3	32.0	34.5
hsa-miR-93-3p	9.70	(9.1-10.28)	10.54	(10.4-11.6)	0.038	30.1	28.3	31.5
hsa-miR-93-5p	16.14	(15.89-16.53)	16.95	(16.71-17.74)	0.021	23.9	22.7	24.4
hsa-miR-99a-5p	10.47	(9.48-10.83)	10.90	(10.35-11.13)	0.105	29.8	29.1	31.4
hsa-miR-99b-5p	10.37	(9.57-11.21)	11.05	(10.34-12.29)	0.130	29.5	28.0	30.8
mmu-miR-378a-3p	11.86	(11.33-12.14)	12.11	(11.56-12.71)	0.328	28.4	27.4	29.3

Data are presented as median (P25-P75). Differences between groups were analyzed using Mann-Whitney U test. Candidates in bold.

Supplemental Table S3. MicroRNA candidates.

	MIRBase ID	Chromosome	Start	End	Strand	Seed+m8	microRNA family
**hsa-miR-15b-3p**	MIMAT0004586	chr3	160404588	160404685	+	GAAUCAU	NA
**hsa-miR-15b-5p**	MIMAT0000417	chr3	160404588	160404685	+	AGCAGCA	NA
**hsa-miR-21-5p**	MIMAT0000076	chr1	59841266	59841337	+	AGCUUAU	miR-21-5p/590-5p
**hsa-miR-22-3p**	MIMAT0000077	chr17	1713903	1713987	-	AGCUGCC	NA
**hsa-miR-27a-3p**	MIMAT0000084	chr19	13836440	13836517	-	UCACAGU	miR-27-3p
**hsa-miR-27b-3p**	MIMAT0000419	chr9	95085445	95085541	+	UCACAGU	miR-27-3p
**hsa-miR-146a-5p**	MIMAT0000449	chr5	160485352	160485450	+	GAGAACU	NA
**hsa-miR-148a-3p**	MIMAT0000243	chr7	25949919	25949986	-	CAGUGCA	miR-148-3p/152-3p
**hsa-miR-148b-3p**	MIMAT0000759	chr12	54337216	54337314	+	CAGUGCA	miR-148-3p/152-3p
**hsa-miR-152-3p**	MIMAT0000438	chr17	48037161	48037247	-	CAGUGCA	miR-148-3p/152-3p
**hsa-miR-339-3p**	MIMAT0004702	chr7	1022933	1023026	-	GAGCGCC	NA
**hsa-miR-590-5p**	MIMAT0003258	chr7	74191198	74191294	+	AGCUUAU	miR-21-5p/590-5p

miRBase database (http://www.mirbase.org/) and Targetscan (http://www.targetscan.org/) were used to describe the microRNAs. NA: Not applicable.

Supplemental Table S4. Correlations between epicardial fat volume and plasma microRNAs.

		miR-15b-3p	miR-15b-5p	miR-21-5p	miR-22-3p	miR-27a-3p	miR-27b-3p	miR-146a-5p	miR-148a-3p	miR-148b-3p	miR-152-3p	miR-339-3p	miR-590-5p
**EFV**	**Spearman's rho**	0.192	0.086	0.054	0.165	0.076	0.125	0.034	0.171	0.160	0.130	0.127	0.141
	**P-value**	0.010	0.251	0.470	0.027	0.308	0.095	0.647	0.022	0.032	0.082	0.090	0.059

EFV: Epicardial fat volume

SUPPLEMENTAL FIGURE S1

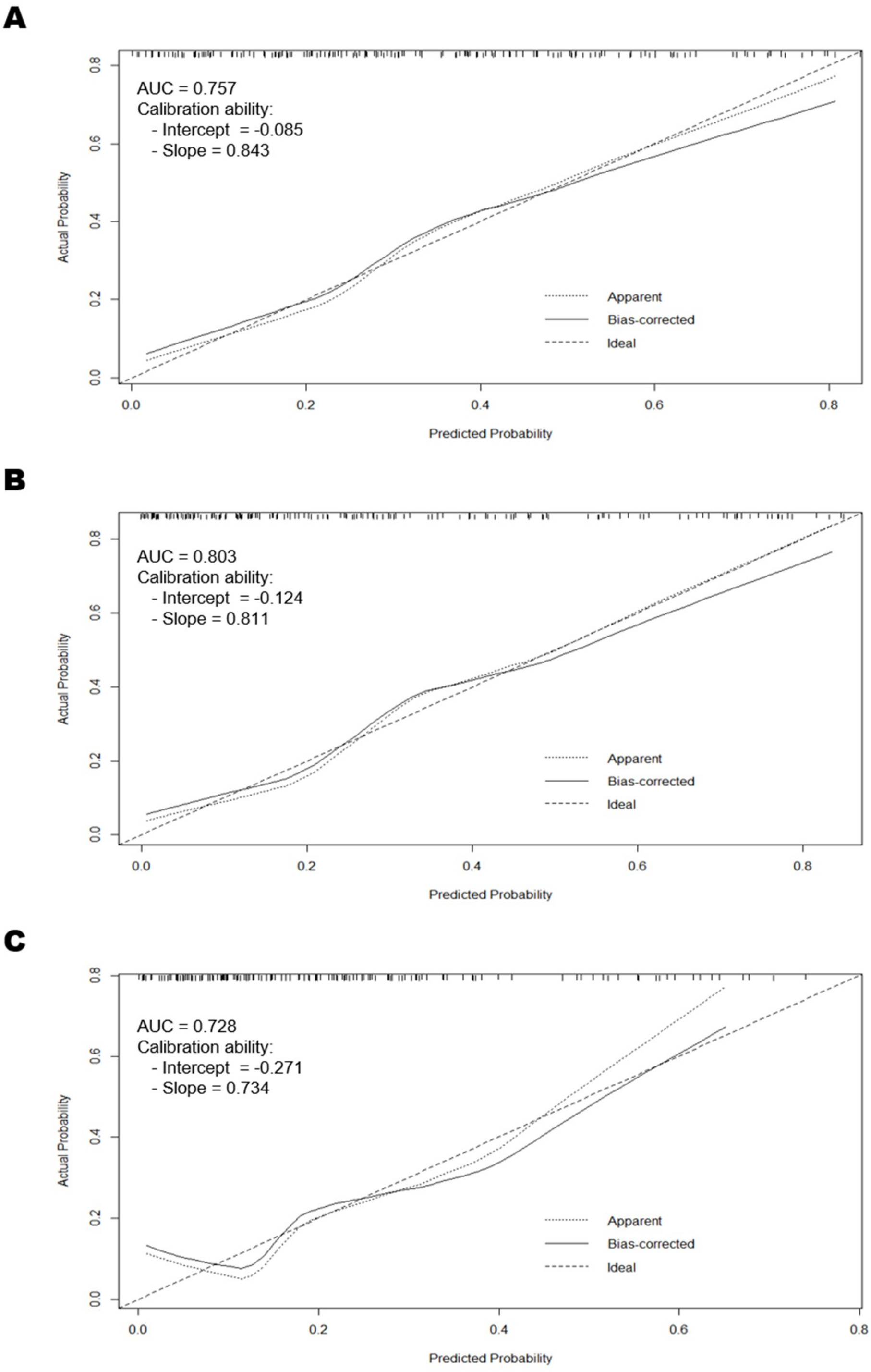

Supplemental Figure S1. Calibration plots (with internal validation). Calibration of the plasma microRNA models for epicardial fat volume tertiles (A), the cutoff proposed by Spearman et al. [15] (B) and the cutoff proposed by Shmilovich et al. [21] (C).

A

Area under the ROC curve of plasma microRNAs.

	AUC	95% CI	P-value
miR-15b-3p	0.615	(0.508-0.722)	0.040
miR-15b-5p	0.535	(0.418-0.653)	0.529
miR-21-5p	0.522	(0.408-0.636)	0.697
miR-22-3p	0.599	(0.486-0.712)	0.077
miR-27a-3p	0.526	(0.401-0.651)	0.643
miR-27b-3p	0.548	(0.421-0.675)	0.388
miR-146a-5p	0.519	(0.398-0.639)	0.737
miR-148a-3p	0.597	(0.487-0.707)	0.082
miR-148b-3p	0.575	(0.454-0.697)	0.179
miR-152-3p	0.583	(0.465-0.701)	0.139
miR-339-3p	0.548	(0.427-0.669)	0.390
miR-590-5p	0.580	(0.465-0.695)	0.153
miR-15b pair	0.634	(0.526-0.742)	0.017
miR-21-5p/590-5p family	0.568	(0.455-0.687)	0.226
miR-27-3p family	0.600	(0.489-0.712)	0.073
miR-148-3p/152-3p family	0.588	(0.478-0.697)	0.118

AUC: Area Under the ROC curve; 95% CI: 95% confidence interval.

B

Performance of plasma microRNAs as biomarkers.

	OR (95% CI)	P-value	AUC (95% CI)	P-value (vs CM)	IDI (95% CI)	P-value	NRI (95% CI)	P-value
**Clinical model**			0.669 (0.576-0.762)					
Age	1.025 (0.992-1.063)	0.152						
Sex	1.490 (0.670-3.472)	0.338						
BMI	1.060 (0.973-1.156)	0.180						
Diabetes mellitus	1.748 (0.688-4.270)	0.227						
**Clinical model + microRNAs**			0.783 (0.685-0.882)	0.025	0.163 (0.094-.0232)	<0.001	0.745 (0.397-10.93)	<0.001
Age	1.023 (0986-1.064)	0.247						
Sex	1.987 (0.826-5.085)	0.136						
BMI	1.070 (0.975-1.175)	0.150						
Diabetes mellitus	1.418 (0.499-3.834)	0.499						
miR-27a-3p	0.179 (0.046-0.623)	0.010						
miR-146a-5p	0.338 (0.126-0.853)	0.025						
miR-148b-3p	3.790 (1.245-14.193)	0.034						
miR-152-3p	0.179 (0.046-0.623)	0.010						

Variables included in the clinical model: Age, sex, body mass index and diabetes mellitus. CM: Clinical model; OR: Odd ratio; 95% CI: 95% confidence interval. AUC: Area Under the ROC curve; IDI: Integrated discrimination improvement; NRI: Net reclassification improvement.

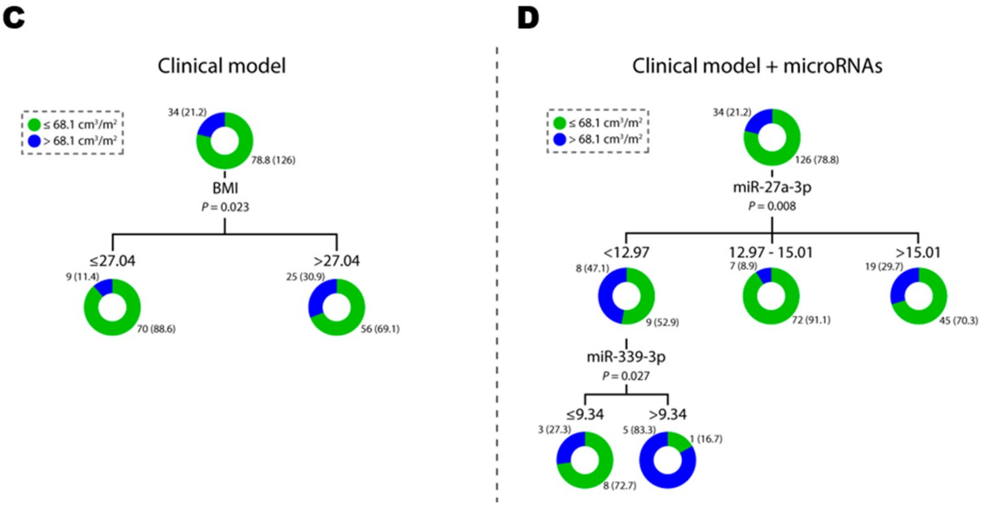

Supplemental Figure S2. Plasma microRNAs (miRNAs) as biomarkers of epicardial fat volume-indexed (EFVi), according to the cutoff proposed by Shmilovich et al. [21]. A) Area under the ROC curve for each individual miRNAs and for combinations of miRNAs in pairs or families. **B)** Performance of plasma miRNAs as biomarkers. **C-D)** Decision trees calculated by Chi-squared Automatic Interaction Detector (CHAID) algorithm. The following variables were included in the clinical model: age, sex, body mass index and diabetes mellitus. MicroRNA levels were log_2-transformed. For logistic regression models, data are presented as an odds ratio (OR) and 95% confidence intervals (CI). For discrimination analysis, data are presented as the AUC and 95% CI. For reclassification analysis, data are presented as the Integrated

Discrimination Improvement (IDI) index and Net Reclassification Improvement (NRI) index and their respective 95% CI. For decision trees, data are shown as frequency (percentage) of patients in each study group.
